# Elevational clines in the temperature dependence of insect performance and implications for ecological responses to climate change

**DOI:** 10.1093/conphys/cou035

**Published:** 2014-08-23

**Authors:** Lauren B. Buckley, César R. Nufio

**Affiliations:** 1 Department of Biology, University of Washington, Seattle, WA 98195-1800, USA; 2 Department of Ecology and Evolutionary Biology, University of Colorado, Boulder, CO 80309, USA; 3 University of Colorado Natural History Museum, University of Colorado, Boulder, CO 80309, USA

**Keywords:** Feeding rate, grasshopper, local adaptation, locomotion, plasticity, thermal performance curve

## Abstract

To what extent is insect hopping and feeding performance, which constrains the ability to obtain and assimilate resources, thermally adapted along an elevation gradient? Does temperature dependence vary between populations and species and can differences account for individualistic responses to past climate change? We investigate these questions for three species of grasshoppers along a Rocky Mountain elevation gradient. All species and populations exhibit warm adaptation for consumption and digestion, with only modest inter- and intra-specific differences. Species differ substantially in the temperature of peak hopping performance. Low-elevation populations of the warm-adapted species exhibit the highest performance at high temperatures and the lowest performance at low temperatures. Developmental plasticity influences the temperature dependence of performance; grasshoppers reared at higher temperatures perform better at higher temperatures and possess broader thermal tolerance. We fitted thermal performance curves to examine whether performance shifts can account for changes in abundance between initial surveys in 1958–1960 and recent surveys since 2006. All species and populations are able to achieve greater feeding rates now. Estimated shifts in hopping performance vary between species and along the elevation gradient. The cool-adapted species has experienced declines in hopping performance, particularly at the lower elevation sites, while the warm-adapted species has experienced increases in performance concentrated at higher elevations. These estimated performance shifts broadly concur with observed abundance shifts. Performance metrics may have a greater potential to elucidate differential responses to climate change between populations and species than coarser and oft-used proxies, such as thermal tolerance. Assessing performance directly when temperature dependence varies between processes such as the acquisition and assimilation of energy may be essential to understanding population- and species-level impacts.

## Abstract (precis)

The temperature dependence of hopping and feeding are locally thermally adapted for three grasshopper species along an elevation gradient. Estimated performance shifts corresponding to recent warming broadly concur with observed abundance shifts. Performance metrics may better predict responses to climate change than coarser and oft-used proxies such as thermal tolerance.

## Introduction

Environmental temperature can constrain ectothermic organisms by limiting their ability to locate, gather, consume and assimilate resources. These processes follow a hump-shaped rate function (thermal performance curve, TPC), with performance being optimal at an intermediate body temperature (Huey and Kingsolver, 1989). Local adaptation in TPCs has been widely observed along elevation and latitudinal gradients (Angilletta, 2009; Kingsolver, 2009), particularly for insects (Hodkinson, 2005). This dependence of performance and fitness on temperature has been used effectively as a basis for understanding and predicting the ecological impacts of climate change on populations and species. For example, Huey *et al.* (2009) predicted declining performance for a cool-adapted tropical lizard in response to climate warming over recent decades. Related analyses suggest that restrictions in lizard performance and activity duration associated with climate change drive extinctions (Sinervo *et al.*, 2010) and range shifts (Buckley *et al.*, 2010; Kearney, 2012).

Latitudinal and altitudinal patterns of thermal specialization and adaptation shape sensitivity to climate change. Deutsch *et al.* (2008) predicted that detrimental effects of climate shifts would be most intense in tropical species with narrow thermal specialization, despite the lesser magnitude of climate change experienced. Subsequent analyses suggest that more variable temperate climates may increase the incidence of thermal stress events and equalize the impacts of climate change across latitudes (Kingsolver *et al.*, 2013). Acclimatization and adaptation of temperature-dependent performance traits is important for understanding future responses to warming climates (Hofmann and Todgham, 2010) and identifying species most vulnerable to change (Huey *et al.*, 2012).

Many analyses of the potential impacts of climate change rely on physiological metrics that are easy to measure, such as critical thermal minima and maxima (CT_min_ and CT_max_); however, the body temperatures of organisms rarely approach CT_min_ and CT_max_ in nature owing to the severe thermal stress experienced at these extreme temperatures. Researchers often use CT_min_ and CT_max_ to estimate TPCs (Angilletta, 2009) and thus responses to more ecologically relevant temperatures, but the ecological significance of CT_min_ and CT_max_ remains questionable.

Phenological and abundance shifts by grasshoppers along a 2000 m elevation gradient near Boulder (CO, USA) in response to climate change over the last 50 years have varied in extent and direction among populations and species (Nufio *et al.*, 2010). Thermal tolerances, thermal preferences and metabolic rate responses varied among species, but inter-population differences were insufficient to account for the individualistic responses to past climate change (Buckley *et al.*, 2013b). Grasshoppers, however, exhibit a pronounced temperature dependence in hopping activity (Harrison *et al.*, 1991) and feeding behaviours (Harrison and Fewell, 1995). Thus, in the present study we test whether TPCs for hopping and feeding differ within and among three species along the above elevation gradient and then consider whether these performance measures can account for the individualistic responses to observed climate change. This addresses whether more refined performance metrics offer greater promise for understanding differential responses to climate change than cruder thermal tolerance metrics.

Finally, we examine the influence of developmental plasticity in shaping TPCs by rearing grasshoppers in a range of constant temperatures. Thermal performance curve are more likely to be developmentally plastic when environmental conductions during development are indicative of those experienced later (Kingsolver and Huey, 1998). Plasticity in thermal traits along elevation gradients are likely to be complex, because high-altitude sites, while generally colder, are more variable, with high levels of radiation producing short periods of high body temperatures (Buckley *et al.*, 2013a). Mean environmental conditions and extreme events combine to influence the position, breadth and shape of TPCs (Schulte *et al.*, 2011). We examine developmental plasticity in TPCs, which provides insight into whether acclimatization of TPCs will contribute to responses to thermal stress.

## Materials and methods

### Study organisms, sites and rearing

We examined three grasshopper species that are expected to differ in their exposure to climate change and in their potential for local adaptation (Table 1). *Melanoplus boulderensis* (part of the *Melanoplus dodgei* species complex; Otte, 2012) is a short-winged species with limited dispersal and high potential for local adaptation. *Camnula pellucida* and *Melanoplus sanguinipes* are, in contrast, long-winged species with a higher dispersal capacity and a greater potential for gene flow across populations. All species occur from the upper foothills to the subalpine. *Melanoplus sanguinipes* and *C. pellucida* are broadly distributed geographically, while *M. boulderensis* is thought to have evolved in the Rocky Mountains within the last million years (Knowles and Otte, 2000). *Melanoplus boulderensis* and *C. pellucida* tend to be cool and warm adapted, respectively, whereas *M. sanguinipes* is a thermal generalist (Buckley *et al.*, 2013b). We previously delineated cool and warm adaptation based on critical thermal limits on activity and preferred body temperatures using field-collected individuals, so we have been unable to exclude developmental and maternal effects. Although maternal effects have been observed for *Melanoplus* species via photoperiod influencing the depth of embryonic diapause (Dean, 1982), we are unaware of documented maternal effects in *Melanoplus* relevant to the thermal metrics we investigated in the present study. Here we present thermal tolerance and performance metrics for laboratory-reared individuals of *M. sanguinipes*. Similarities in these metrics between field-collected and laboratory-reared individuals support the hypothesis of local adaptation across species, but further work to partition genetic and plastic effects on phenotypes will be required to establish local adaptation. All three species consume a variety of forbes and grasses.

**Table 1:** Overview of the focal grasshopper species and observed shifts in their phenology and abundance between 1959–1960 and 2006–2008 (data from Nufio *et al.*, 2010; Buckley *et al.*, 2013b)

Species	Dispersive	Thermal adaptation	Seasonal timing	Phenology (↓ earlier)			Abundance		
				2195 m	2591 m	3048 m	2195 m	2591 m	3048 m
*Melanoplus boulderensis*	No	Cool	Early	↓	↓	≈	↓	↓	≈
*Camnula pellucida*	Yes	Warm	Middle	↑	↓	↓	↓	↓	↑
*Melanoplus sanguinipes*	Yes	Generalist	Late	↑	↑	^a^	↓	↓	^a^

^a^Low abundance during initial time period prevents comparison.

We examined variation in thermal performance curves among populations and species across five sites along the following elevation gradient: Red Fox (1574 m, 40.05N, 105.19W), A1 (2195 m, 40.01N, 105.37W), B1 (2591 m, 40.02N, 105.43W), C1 (3048 m, 40.03N, 105.55W) and D1 (3739 m, 40.06N, 105.62W; Alexander and Hilliard, 1969; descriptions can be found at http://niwot.colorado.edu/site_info/site_info.html). The sites are grassy clearings associated with upper prairie, foothill, montane, subalpine and alpine life zones, respectively. Adults from populations at each site were kept separately with access to organic romaine lettuce and wheat bran and acclimated overnight in an incubator to an ‘intermediate’ temperature of 25°C. Individuals were then transferred to polyurethane containers for assessment of their hopping performance and feeding rate. When not undergoing tests they were maintained in the same conditions described above.

In order to examine how plasticity influences the temperature dependence of performance, we repeated the performance assessments for *M. sanguinipes* reared at constant 24 or 27°C and a 14 h–10 h light–dark cycle in Percival I-36VL incubators. We reared the other species and, additionally, *M. sanguinipes* at 18 and 30°C, but low hatching, development or survival rates prevented analysis. Eggs were collected the previous summer by allowing females to oviposit in damp sand and then sieving the sand to collect the egg pods. Egg pods were then placed in damp vermiculite within condiment containers. The surface was periodically coated with 0.25% methyl-*p*-hydroxy benzoate to inhibit fungal or microbial growth. The eggs were allowed to develop for 3 weeks at 25–30°C and were then stored at 2°C for ∼110 days to enable diapause. The egg containers were subsequently placed in a 24 or 27°C incubator. Upon hatching, the egg containers were placed within rectangular 2.25 l polyurethane containers, and romaine lettuce and wheat bran were provided. The grasshoppers were reared together until they reached third instar. Subsequently, grasshoppers were reared individually in 0.47 l polyurethane containers, which were changed every other day and supplied with romaine lettuce and wheat bran. Adults were acclimated to 25°C before commencing the performance assessments.

### Temperature dependence of performance

Grasshoppers were fasted prior to each feeding trial for 12 h, a sufficient period to complete digestion and absorption (Harrison and Fewell, 1995), and provided with a damp paper towel for humidity. The feeding trials were conducted at six temperatures (15, 20, 25, 30, 35 and 40°C; temperature range after Harrison and Fewell, 1995). Trials at two temperatures were run concurrently each day, with individuals from each population randomly partitioned between the two temperatures. We assessed performance for an average of 20 individuals from each population (range 14–22). The order of temperature trials was randomized. Grasshoppers were acclimated to the test temperature for 1 h prior to being provided with organic, baby romaine leaves at the start of two consecutive feeding periods each day. The first feeding period lasted for 2 h (reflecting rates of ingestion and of crop and mid-gut filling) and the later feeding period lasted an additional 6 h (reflecting rates of ingestion, crop filling and gut throughput; Harrison and Fewell, 1995). The initial feeding trials commenced between 07.00 and 09.00 h. We used a flatbed scanner (Canon LiDE 100) to photograph the leaves before and after each of the feeding trials. We estimated leaf areas using ImageJ software (http://rsbweb.nih.gov/ij/).

In order to assess the temperature dependence of hopping performance, grasshoppers were acclimated for 1 h at one of four possible temperatures (10, 17, 25 or 35°C). Grasshoppers were then removed individually from the incubators and immediately placed in the centre of the experimental arena at room temperature. The arena consisted of a 1.8 m × 1.8 m sheet of fabric with a checkered pattern at an interval of 2.5 cm (methods follow Harrison *et al.*, 1991). Hopping was induced by manual prodding if necessary. We marked the position of the grasshopper after each of five jumps and subsequently recorded the *x* and *y* locations to an *x* and *y* resolution of 2.5 cm. We used the means of the five jumps for analysis, but checked that results were similar when using the median. Each individual was tested at each of the four temperatures, with the order of temperatures randomized. Performance of *C. pellucida* was observed to be minimal at the lowest temperature (10°C) and was thus not assessed.

For reared *M. sanguinipes* individuals, we measured both CT_min_ and CT_max_, which were defined as the lower and upper temperatures at which the grasshoppers were no longer able to right themselves. Grasshoppers were placed individually into 50 ml centrifuge tubes, which were slowly (∼0.2°C min^−1^) cooled or heated in a water bath. Given that warming rates may influence estimates of critical thermal limits, we chose an intermediate rate of warming (Chown *et al.*, 2009). To minimize stress, we first cooled body temperatures and then began heating body temperatures after an hour.

We used maximum likelihood to fit linear mixed-effects models describing feeding and hopping rates as a function of species, elevation, temperature and their interaction (function lme in R). We accounted for repeated measures of individuals by including an individual identifier as a random variable. For the reared grasshoppers, we additionally included a clutch identification as a random variable in which the individual identification was nested. We subsequently dropped the clutch identification as a random effect because its inclusion did not influence our results qualitatively.

### Thermal performance curves

We fitted TPCs to our hopping and feeding performance data to determine the degree to which performance may have shifted through recent climate change along the elevation gradient. We fitted TPCs as a product of a Gaussian function and a Gompertz function (Frazier *et al.*, 2006), as follows:

$$P(T)=P_{\max}e^{-e[\rho (T-T_{o})-6]-\sigma (T-T_{o}{)}^{2}},$$

where *P*(*T*) is the feeding rate at temperature *T*, *P*_max_ is the maximal performance rate, *T*_o_ is the optimal temperature, and *ρ* and *σ* determine the thermal sensitivity of performance at temperatures above and below *T*_o_, respectively. We estimated parameters using the nls2 function in R. We assumed that *ρ* = 0.7, because we had limited data with which to fit the upper portion of the performance curve.

We assumed that the *T*_o_ was within 3°C of each species' preferred body temperature (Buckley *et al.*, 2013b) because the upper portion of the performance curve was poorly characterized by our data, particularly for *C. pellucida*. Additionally, we accounted for the minimal performance of *C. pellucida* at 10°C by including data points corresponding to zero performance at the CT_min_ (species mean ± SD = 9.33 ± 1.05°C; Buckley *et al.*, 2013b). The number of these data points was set at a quarter of the number of individuals included in the performance assay to avoid unduly influencing the TPC fit. Temperature values (*x*-axis) were drawn from a normal distribution based on the mean and standard deviation of CT_min_ for each population of *C. pellucida* (Buckley *et al.*, 2013b).

### Biophysical model and weather data

We estimated performance shifts over the past 50 years at our study sites based on predictions of grasshopper body temperature. We used a steady-state budget of energy flux to describe the flow of energy between grasshoppers and their environments and predict operative body temperature, *T*_e_, as follows: *Q*_s_ * = * *Q*_t_ * + * *Q*_c_ * + * *Q*_cond_. Here *Q*_s_ is the total input of heat flux due to solar radiation; *Q*_t_ describes the flux of thermal radiative heat due to both incoming thermal radiation (ground and sky) and that emitted by the grasshopper; *Q*_c_ is the heat flux between the grasshopper and the surrounding fluid (air) via convection; and *Q*_cond_ is the heat flux between the grasshopper's body and the solid surfaces with which the grasshopper's body is in contact via conduction. We used this energy flux model to solve for *T*_e_, because rates of thermal radiative heat flux, convection and conduction are functions of *T*_e_. We omitted evaporative heat loss because it is negligible for grasshoppers in most conditions (Anderson *et al.*, 1979). The detailed description and validation of our biophysical model for grasshoppers is given elsewhere (Anderson *et al.*, 1979; Buckley *et al.*, 2013b). We assumed the mean body size among our species to be 21 mm. Preliminary analysis of body size trends over time suggest that any body size shifts have been modest. We also assumed that the absorptivity of the grasshopper's body is 0.7 and the albedo of the substrate is 0.3 (Kingsolver, 1983). These parameters determine the rate of energy exchange between the grasshopper and its environment.

Many insects, including grasshoppers, use thermoregulatory behaviours to buffer environmental variation along altitudinal gradients (Chappell and Whitman, 1990; Samietz *et al.*, 2005). We bracketed the range of potential body temperatures by assuming that grasshoppers can move between full sun and full shade (no direct radiation). We also assumed that the grasshoppers can move between the ground (25% contact of surface area) and vegetation (0% contact of surface area). This assumption determines rates of conduction between the ground and the grasshopper. We assumed that individuals of each species thermoregulate to the available body temperature closest to the species' preferred body temperature (Buckley *et al.*, 2013b). We examined sensitivity to the assumptiuon of thermoregulation by additionally considering grasshoppers in vegetation (no conduction with the ground) that are unable to thermoregulate.

We parameterized the biophysical model with daily maximum and minimum temperature data from weather stations at our study sites (McGuire *et al.*, 2012). We estimated hourly air temperatures (*T*_a_) using a diurnal temperature variation function based on maximum and minimum temperatures and the Julian date as modelled by Parton and Logan (1981). Daytime temperatures were fitted using a sine wave, while night-time temperatures were modelled using an exponential function. We assumed that ground temperatures were 8.4°C above *T*_a_ during daylight hours (Kingsolver, 1983). We predicted global solar radiation at the ground surface (in watts per square metre) as a function of latitude and altitude using an algorithm by Nikolov and Zeller (1992).

## Results

### Temperature dependence of feeding

**Figure 1:**
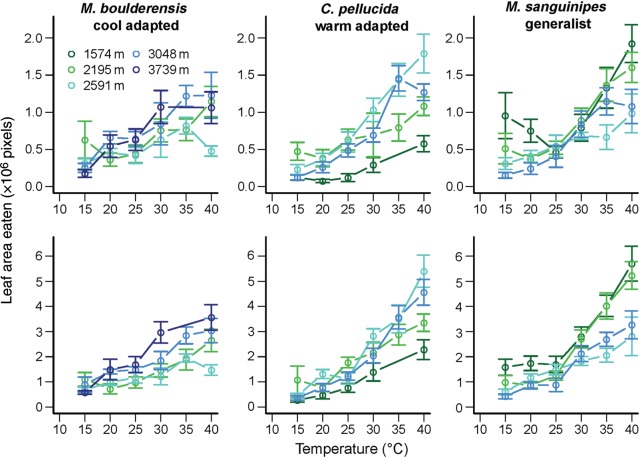
Grasshopper feeding rates (×10^6^ pixels) over a 2 (top panels) or 8 h period (bottom panels) as a function of temperature (in degrees Celsius) for field-collected grasshoppers. Mean values ± SEM are depicted, with colours distinguishing sites.

Grasshopper feeding rates increased with increasing temperature over the range of body temperatures chronically realized among our study sites (15–40°C; Fig. 1). Temperature was the primary predictor of (mass-corrected) feeding rates over a 2 h period in an ANOVA also including species, population elevation and all interactions (Table 2). The effect of temperature on feeding rate differed significantly between species and as a function of elevation. The effect of elevation on feeding rate differed significantly between species. The three-way interaction between temperature, species and elevation was also significant. We omitted sex from this and subsequent models because it did not have a significant effect. The significant predictors of feeding rate were similar over an 8 h period, with temperature remaining the primary predictor (Table 2). In ANOVAs with only main effects, the rate of increase in feeding rate with temperature was similar between the 2 and 8 h periods (mean ± SEM = 0.037 ± 0.002 vs. 0.030 ± 0.001 h pixel^−1^ °C^−1^, respectively, when normalized to a 2 h period). This suggests that rates of consumption and assimilation have similar temperature dependence.

**Table 2:** Results of ANOVAs examining the determinants of grasshopper feeding rates (×10^6^ pixels) over a 2 or 8 h period

Parameters	2 h *F*_df,407_	8 h *F*_df,407_
Temperature (°C)	254.8***	651.6***
Species	4.6*	8.4***
Elevation (m)	0.2	1.0
Temperature × species	3.9*	21.1***
Temperature × elevation	8.4**	5.1*
Species × elevation	16.8***	25.1***
Temperature × species ×elevation	4.2*	15.3***

Significance levels are depicted (*P < 0.05, **P < 0.01 and ***P < 0.001).

The temperature dependence of feeding varied between species. While populations of *M. sanguinpes* exhibited limited feeding at low temperatures over 8 h, the low-elevation populations tended to capitalize best on warm temperatures (elevation, *F*_1,63_ = 14.1, *P* = 0.0004; temperature, *F*_1.39_ = 58.7, *P* < 0.0001; and temperature × elevation, 0.003 ± 0.002, *F*_1,39_ = 6.0, *P* = 0.02, in an ANOVA for temperatures > 25°C). In contrast, high-elevation populations of the cool-adapted *M. boulderensis* exhibited higher feeding rates over 8 h across temperatures (elevation, −0.0006 ± 0.0003, *F*_1,72_ = 11.5, *P* = 0.001; temperature, *F*_1,130_ = 126.7, *P* < 0.0001; and temperature × elevation, *F*_1,130_ = 13.8, *P* = 0.0003 in an ANOVA).

### Temperaure dependence of hopping

**Figure 2:**
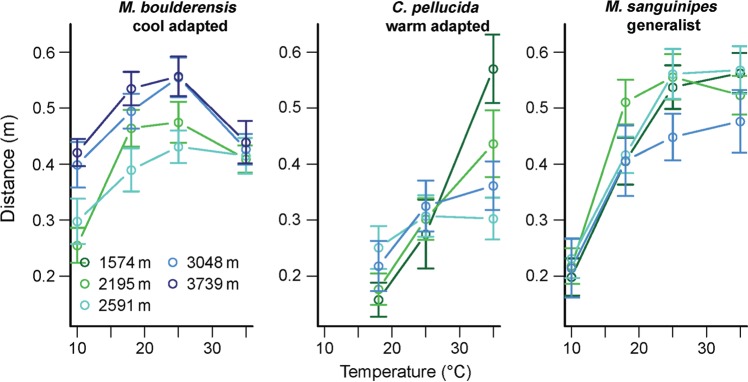
Hopping distance (in metres) as a function of temperature (in degrees Celsius) for field-collected grasshoppers. Mean values ± SEM are depicted, with colours distinguishing sites.

The temperature dependence of hopping performance varied between populations and species (Fig. 2). Temperarature was the primary predictor of hopping distance (temperature included as a polynomial, *F*_1,619_ = 152.5, *P* < 0.0001) in an ANOVA also including species (*F*_2,230_ = 44.7, *P* < 0.0001), source elevation of the population (*F*_1,230_ = 0.7, *P* = 0.4) and all interactions. The effect of temperature on hopping distance differed significantly between species (*F*_2,619_ = 27.5, *P* < 0.0001) and as a function of elevation (*F*_1,619_ = 18.2, *P* < 0.0001). The effect of elevation on hopping distance differed significantly between species (*F*_2,230_ = 3.5, *P* = 0.03). The three-way interaction between temperature, species and elevation was also significant (*F*_2,619_ = 5.5, *P* = 0.004). We omitted mass from this and subsequent models because it did not have a significant effect. We additionally omitted sex, which had a weakly significant main effect but did not alter the models qualitatively.

We next used ANOVAs for each species to explore further how the temperature dependence of hopping varied between species. The cool-adapted *M. boulderensis* exhibited peak performance at lower temperatures than the other species (Table 3 and Fig. 2). High-elevation populations exhibited higher performance. Accounting for mass differences between populations in the ANOVA did not account for the elevation differences (mass, *F*_1,72_ = 1.2, *P* = 0.3). The temperature dependence varied as a function of source elevation for *M. boulderensis*. Populations of the warm-adapted *C. pellucida* also differed in their temperature dependence, with grasshoppers from the low-elevation sites tending to exhibit the strongest temperature dependence (Table 3 and Fig. 2), i.e. the low-elevation populations exhibited the highest performance at high temperatures and the lowest performance at low temperatures. Our trial temperatures were generally lower than the thermal optima for *C. pellucida*. *Melanoplus sanguinipes* tended to be a thermal generalist, with low performance at low temperatures but otherwise limited temperature dependence. The temperature dependence did not vary between populations (Table 3).

**Table 3:** Results of ANOVAs examining the determinants of grasshopper hopping distances (in metres)

Parameters	*M. boulderensis F*_df,221_	*C. pellucida F*_df,162_	*M. sanguinipes F*_df,230_
Temperature (°C)	13.5***	63.2***	167.6***
Temperature^2^ (°C)	56.0***	0.3	44.8***
Elevation (m)	12.2***	0.7	0.4
Elevation × temperature	6.2*	19.7***	1.6
Elevation × temperature^2^	0.0	2.5	0.2

Significant levels are depicted (*P < 0.05, ***P < 0.001).

### Shifts in performance over time

**Figure 3:**
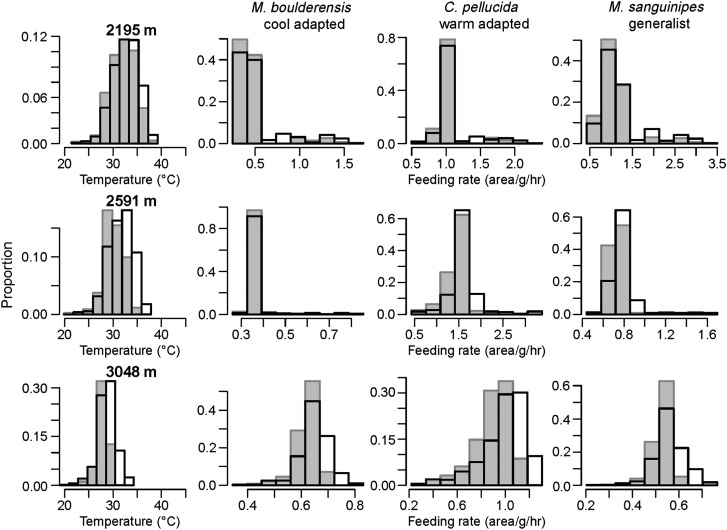
Histrogram depictions of estimated feeding performance shifts corresponding to the changes in daily mean temperature (left panels) at sites along the elevation gradient. We compare 1955–1965 (grey bars) with 2000–2010 (open bars). Estimated shifts in feeding rates (area g^−1^ h^−1^) are depicted for the three species. Values are averaged across days over daylight hours between 15 June and 15 August.

Estimated shifts in performance between 1955–1965 and 2000–2010 varied between species and along the elevation gradient (Fig. 3). Parameters for the fitted TPCs are compiled in Supplementary material, Table S1. Climate change in the interim has been more pronounced at higher elevations. We estimated that all species have experienced an increase in feeding rate in response to recent climate change. Predicted increases have been greater at higher elevations for *C. pelludica* and at lower elevations for *M. boulderensis* and *M. sanguinipes* (change in feeding rate for *M. boulderensis*, 2195 m = 10.7%, 2591 m = 5.6% and 3048 m = 4.7%; for *C. pellucida*, 2195 m = 5.4%, 2591 m = 11.5% and 3048 m = 10.7%; and for *M. sanguinipes*, 2195 m = 10.0%, 2591 m = 9.0% and 3048 m = 6.7%). We additionally considered shifts in feeding rate for a grasshopper on vegetation unable to thermoregulate. In this case, augmentation of feeding rates increased with elevation for all species (Supplementary material, Fig. S1). We noted that these increases are likely to be overestimates because we did not detect an upper temperature at which feeding rate declined in our feeding trials.

**Figure 4:**
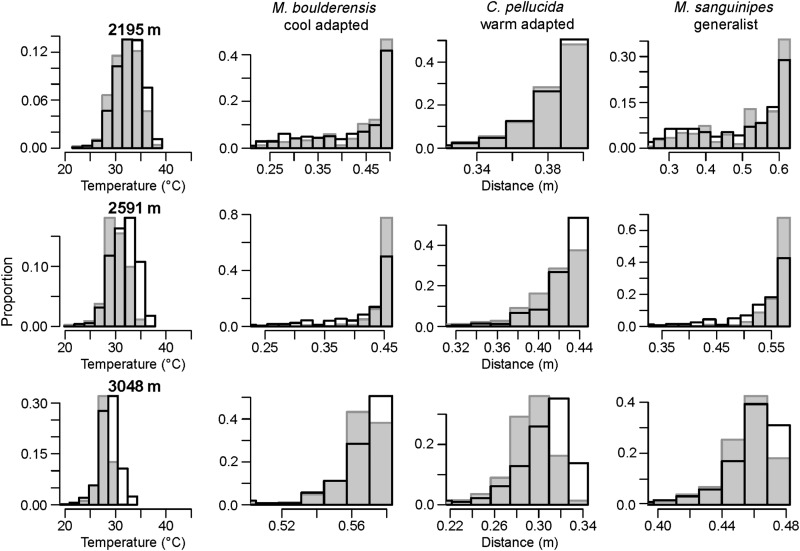
Histrogram depictions of estimated hopping performance shifts corresponding to the changes in daily mean temperature (left panels) at sites along the elevation gradient. We compare 1955–1965 (grey bars) with 2000–2010 (open bars). Estimated shifts in distance (in metres) are depicted for the three species. Values are averaged across days over daylight hours between 15 June and 15 August.

We estimated that the cool-adapted *M. boulderensis* has experienced hopping performance declines at the lower elevation sites (change in performance, 2195 m = −3.8%, 2591 m = −7.0% and 3048 m = 0.0%; Fig. 4). We estimated that the warm-adapted *C. pellucida* has experienced performance increases concentrated at higher elevations (2195 m = 0.5%, 2591 m = 2.2% and 3048 m = 4.3%). We estimated that *M. sanguinipes* has experienced hopping performance declines at lower elevations and a weak performance increase at the highest elevation (2195 m = −4.5%, 2591 m = −5.3% and 3048 m = 0.6%). In the case of a grasshopper on vegetation unable to thermoregulate, we estimated that performance declines are most pronounced at mid-elevation for the warm-adapted *C. pellucida* and at mid- to high elevation for the other species (Supplementary material, Fig. S2).

### Importance of plasticity

**Figure 5:**
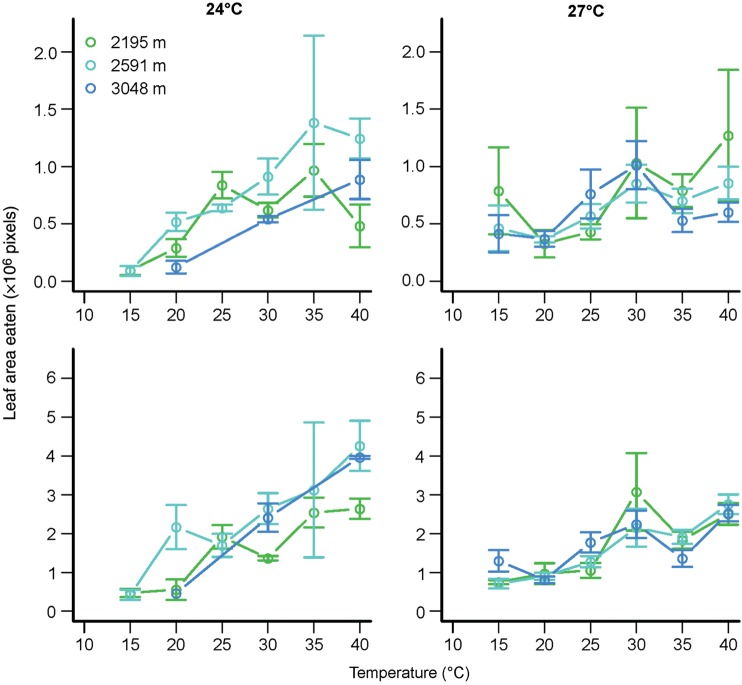
Grasshopper feeding rates (×10^6^ pixels) over a 2 (top panels) or 8 h period (bottom panels) as a function of temperature (in degrees Celsius) for *M. sanguinipes* reared in the laboratory at 24 (left panels) and 27°C (right panels). Mean values ± SEM are depicted, with colours distinguishing sites.

We repeated the performance assays for *M. sanguinipes* reared in constant conditions to examine the extent to which developmental plasticity influences the temperature dependence of performance. Neither the main effect of rearing temperature (*F*_1,179_ = 1.43, *P* = 0.2) nor its interaction with temperature was a significant predictor of feeding rate over a 2 h period in an ANOVA including temperature, elevation, rearing temperature and all interactions (Fig. 5). Only the main effect of temperature was significant. However, plasticity did appear to influence feeding rate over an 8 h period. Rearing temperature exhibited a significant main effect (*F*_1,179_ = 107.4, *P* < 0.0001), a significant interaction with elevation (*F*_1,179_ = 4.7, *P* = 0.03) and a non-significant but suggestive interaction with assessment temperature (*F*_1,179_ = 2.9, *P* = 0.09) as a predictor of feeding rate over an 8 h period in an ANOVA including temperature, elevation, rearing temperature and all interactions. No other terms were significant. This suggests that the temperature dependence of assimilation is more plastic than the temperature dependence of consumption.

**Figure 6:**
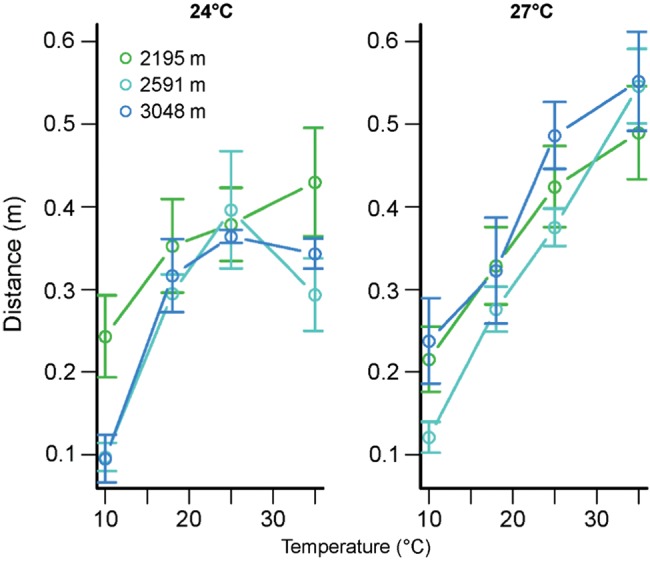
Hopping distance (in metres) as a function of temperature (in degrees Celsius) for *M. sanguinipes* reared in the laboratory at 24 (left panel) and 27°C (right panel). Mean values ± SEM are depicted, with colours distinguishing sites.

We found more evidence for plasticity in the temperature dependence of hopping. Across three populations, we found that individuals reared at 27°C exhibited higher hopping performance at high temperatures than did individuals reared at 24°C (Fig. 6). We found strong temperature dependence (with temperature included as a polynomial, *F*_1,262_ = 142.4, *P* < 0.0001) in an ANOVA with elevation (*F*_1,262_ = 0.2, *P* = 0.7), rearing temperature (*F*_1,262_ = 4.7, *P* = 0.03) and all interactions (temperature × elevation, *F*_1,262_ = 1.2, *P* = 0.3; and three-way interaction, *F*_1,262_ = 0.0, *P* = 1). In addition to the significant main effect of rearing temperature, rearing temperature altered the temperature dependence of hopping (interaction, *F*_1,262_ = 8.2, *P* = 0.005).

We measured CT_min_ and CT_max_ for the reared grasshoppers to assess further the influence of plasticity on thermal tolerance. We found that CT_min_ was significatly lower (mean ± 95% confidence interval for 24°C = 9.38 ± 0.39 and for 27°C = 8.15 ± 0.27; *F*_2,64_ = 2816, *P* < 0.0001) and CT_max_ significantly higher (24°C = 53.9 ± 0.76 and 27°C = 58.7 ± 0.54; *F*_2,64_ = 32 305, *P* < 0.0001) for the grasshoppers reared at 27°C compared with those reared at 24°C. Thermal tolerances did not differ significantly between populations from different elevations. The CT_min_ (8.49 ± 0.35°C) and CT_max_ (57.52 ± 0.31°C) of field-collected *M. sanguinipes* were intermediate between the values for the laboratory-reared grasshoppers (Buckley *et al.*, 2013b).

## Discussion

Our analyses suggest that performance metrics offer greater promise for understanding differential responses to climate change than cruder thermal tolerance metrics. Our focal grasshoppers have extremely high CT_max_ (>57°C; Buckley *et al.*, 2013b), presumably due to the need to tolerate high body temperatures associated with radiation spikes at altitude. We did not detect upper thermal limits for feeding performance and did not fully assess those for hopping performance, but our data suggest that upper limits occur at temperatures substantially below CT_max_. We previously found only limited differences in critical thermal tolerances and preferred body temperatures between the grasshopper species and populations (Buckley *et al.*, 2013b). These previously observed differences were insufficient to account for differential responses to recent climate change. Although CT_min_ and CT_max_ reflect thermal limits for activity as measured in the laboratory, the thermal range for activity tends to be more thermally restricted in the field (Huey, 1982). The CT_max_ reflects the ability to withstand thermal extremes and is more closely predicted by temperature variation than mean temperature (Clusella-Trullas *et al.*, 2011). The CT_min_ reflects both thermal means and extremes (Clusella-Trullas *et al.*, 2011). Another limitation is that estimates of critical thermal limits are sensitive to methology (Chown *et al.*, 2009). The relevance of thermal tolerances to climate-change responses is dependent on whether individual fitness and population dynamics tend to be more limited by acute thermal stress or by chronic energetic conditions (Huey, 1982). The limiting mechansism is related to the distance between limits on activity and lethal limits and how this distance compares with environmental variability (Woodin *et al.*, 2013). Thermal tolerances are likely to be better predictors for populations near latitudinal and elevational range limits, where individuals may be more likely to encounter stressful temperatures (Bozinovic *et al.*, 2011).

We find that grasshoppers are adapted to feed at warm temperatures even in cool, alpine environments. This is consistent with grasshoppers being effective thermoregulators, able to achieve high body temperatures (Harrison and Fewell, 1995; Lactin and Johnson, 1995) even at altitude. Using field-collected individuals, we found similar temperature dependence for food processing (8 h measurements) and consumption (2 h measurements). Temperature sensitivities (*Q*_10_) were greater for *C. pelludica* and *M. sanguinipes* (0.8–1.1) than for the cool-adapted *M. boulderensis* (∼0.4). In contrast to our results, food processing was found to be more temperature sensitive (*Q*_10_) than chewing and crop filling rates for a low-elevation population of another Colorado grasshopper species (Harrison and Fewell, 1995).

Grasshopper hopping performance, which corresponds to the ability to locomote for foraging and escape from predators, does not exhibit the same warm adaptation as feeding. These thermal constraints differ from those on vertebrate ectotherms, whose TPCs for assimilation tend to be narrower than those for locomotion (Angilletta, 2009). Due to the broader TPCs for assimilation than those for locomotion for grasshoppers, we estimate that recent climate change has driven declines in hopping performance but an increased capacity for feeding for species other than the warm-adapted *C. pellucida* at most sites. We expect that grasshoppers will face restrictions in activity time available for foraging, but that they will readily be able to consume and assimilate the vegetation obtained. What will these counteracting shifts mean for energetics and population dynamics? Integrating their effects is challenging because grasshoppers are likely to partition these activities temporally; they may forage during the cool mornings and then rapidly digest food during warm afternoons (Harrison and Fewell, 1995).

Do our expectations for performance shifts align with observed abundance changes in the sites between 1959–1960 and 2006–2008? Abundance has declined for the three focal species at the lower elevation sites. At the highest elevation site, *C. pellucida* abundance has increased and *M. boulderensis* abundance has remained approximately constant since initial surveys. *Melanoplus sanguinipes* was absent or at low abundance at the highest elevation site during the initial surveys (Fig. S1 of Buckley *et al.*, 2013b; Table 1). We estimate hopping performance increases concentrated at high elevation for the warm-adapted *C. pellucida* since the initial surveys. We estimate that *M. boulderensis* experienced hopping performance declines at the lower elevation sites and no change in hopping performance but increased feeding performance at the high-elevation site. We estimate performance declines at lower elevations but a weak increase at the high-elevation site for *M. sanuinipes*. These estimates roughly correspond to the observed abundance shifts in response to recent climate change.

Our findings highlight the potential importance of plasticity to responses to climate change (Chevin *et al.*, 2010). The greater plasticity of hopping relative to feeding may result from all populations and species being selected to take advantage of the warm temperatures available across elevations by basking in sunny areas for feeding and assimilation. However, we find that the assimilation rates of high-elevation populations increase with temperature more strongly at the lower rearing temperature. This suggests the importance of taking advantage of high temperatures for assimilation when they are available in cold environments. The greater plasticity of assimilation compared with consumption may stem from differences in the potential for enzymatic alterations. Likewise, rearing at warmer temperatures increases hopping performance at high temperatures, providing additional evidence that organisms take advantage of warm temperatures. This plasticity is consistent with a hotter-is-better dynamic (Angilletta, 2009).

The observed plasticity in hopping performance may mitigate some of the performance declines predicted to result from climate change. Indeed, plasticity has played a substantial role in recent responses, with most evidence coming from changes in body size resulting from shifts in growing season and survival (Ozgul *et al.*, 2009, 2010) and changes in breeding (Réale *et al.*, 2003; Charmantier *et al.*, 2008) and flowering phenology (Anderson *et al.*, 2012). Plasticity will both enable rapid responses to climate change and buffer the selection potentially required to cope with climate change in the long term (Chevin *et al.*, 2010). Plasticity can substantially enhance the rate of climate change that organisms can pace (Gienapp *et al.*, 2013).

While less is known about how thermal acclimatization has contributed to recent responses to climate change, thermal acclimatization in response to extreme temperatures—such as cold hardening—is well documented (Sinclair *et al.*, 2003; Angilletta, 2009). Both short-term reversible acclimatization and developmental acclimatization will be likely to be important to responses to climate change (Angilletta, 2009). Less is known about the potential for developmental acclimatization because few laboratory studies have examined development and fitness in realistically variable environments (Kingsolver *et al.*, 2013). Further examination of developmental plasticity in variable environments would refine our understanding of how plasticity may alleviate performance declines resulting from climate change.

## Supplementary material

Supplementary material is available at *Conservation Physiology* online.

Supplementary Data
