# Supplementary material for: Elevational clines in the temperature dependence of insect performance and implications for ecological responses to climate change
Source: Conserv Physiol. 2014 Aug 23;2(1):cou035. doi: 10.1093/conphys/cou035 (PMC4806720; doi:10.1093/conphys/cou035)
Supplement: Supplementary Data [file supp_2_1_cou035__index.html]

Supplementary Data 

# Elevational clines in the temperature dependence of insect performance and implications for ecological responses to climate change

## Supplementary Data

Supplementary Data

**Files in this Data Supplement:**

- Supplementary Data - Docx file
