# Supplementary material for: Elevational clines in the temperature dependence of insect performance and implications for ecological responses to climate change
Source: Conserv Physiol. 2014 Aug 23;2(1):cou035. doi: 10.1093/conphys/cou035 (PMC4806720; doi:10.1093/conphys/cou035)
Supplement: Supplementary Data [file supp_cou035_cou035supp.docx]

**Supplementary Data**

**Table S1**: Parameter estimates for thermal performance curves for hopping rate fitted as a product of a Gaussian function and a Gompertz function, where *P*_max_ is the maximum performance rate (m), *T*_o_ is the optimal temperature (°C), and σ determines the thermal sensitivity of performance (m/°C) at temperatures below *T_o_* (Frazier *et al.*, 2006).





**
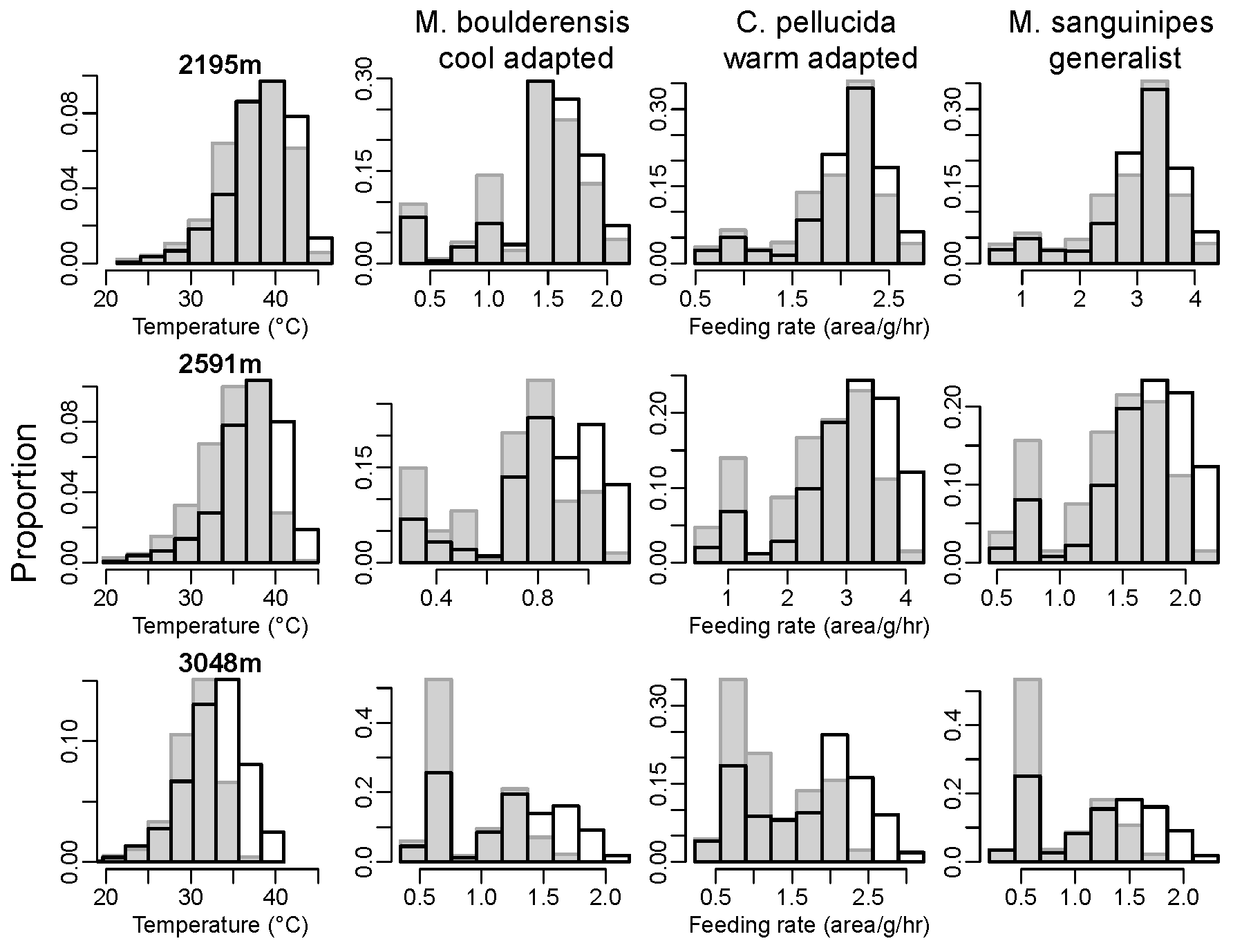
**

**Figure S1**: Histrogram depictions of estimated feeding performance shifts corresponding the changes in daily mean temperature (left) at sites along the elevation gradient. These estimated performance shifts correspond to no thermoregulation or conduction with the ground. We compare 1955-1965 (gray bars) to 2000-2010 (hollow bars). Estimated shifts in feeding rates (area g^-1^ hr^-1^) are depicted for the three species. Values are averaged across days over daylight hours between June 15 and August 15.


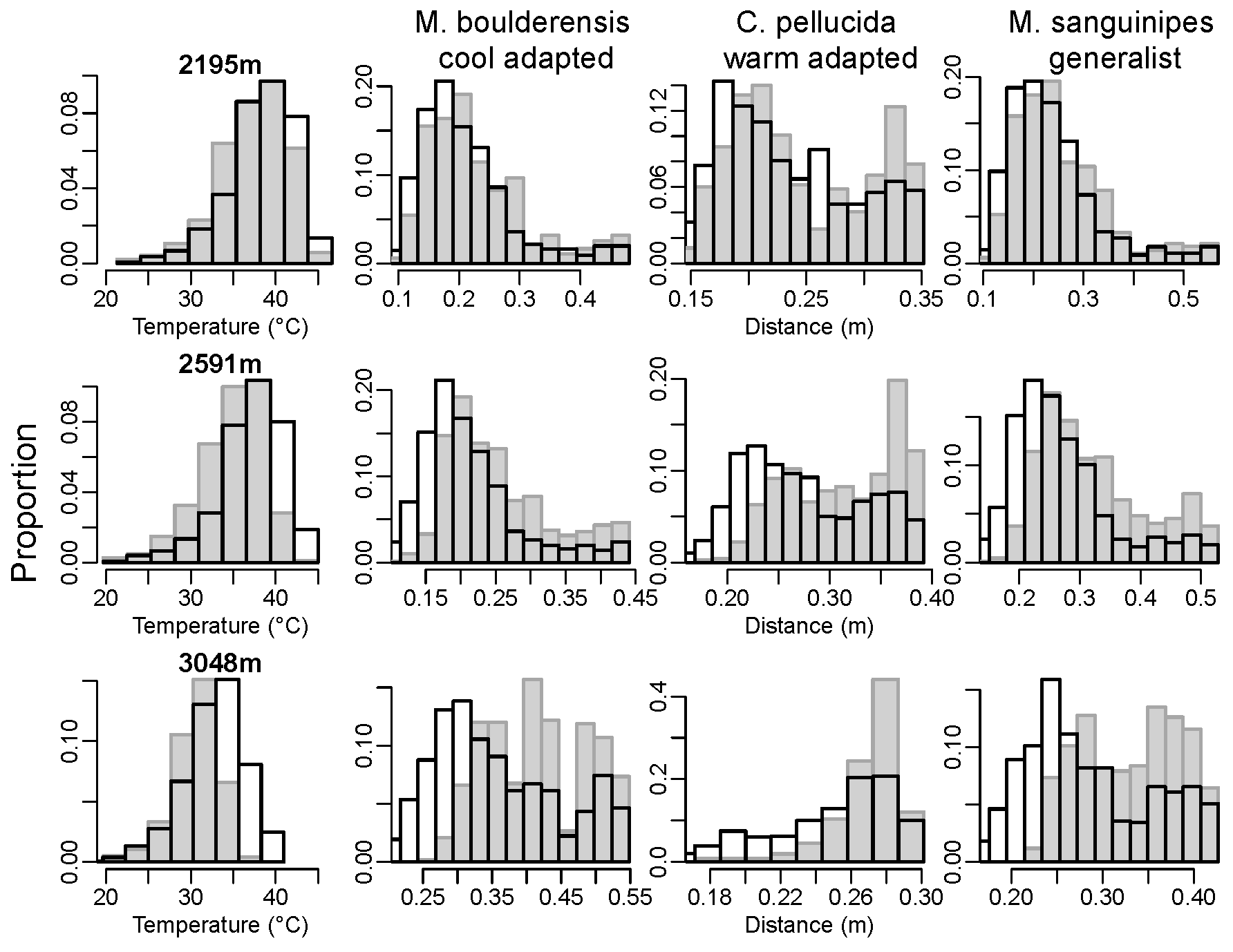


**Figure S2**: Histrogram depictions of estimated hoping performance shifts corresponding the changes in daily mean temperature (left) at sites along the elevation gradient. These estimated performance shifts correspond to no thermoregulation or conduction with the ground. We compare 1955-1965 (gray bars) to 2000-2010 (hollow bars). Estimated shifts in distance (m) are depicted for the three species. Values are averaged across days over daylight hours between June 15 and August 15.
